# Left ventricular hypertrophy and myocardial fibrosis in heart failure with preserved ejection fraction: mechanisms and treatment

**DOI:** 10.1093/eurheartj/ehaf524

**Published:** 2025-09-02

**Authors:** Alexander Peikert, Marianna Fontana, Scott D Solomon, Thomas Thum

**Affiliations:** Department of Cardiology, University Heart Center Graz, Medical University of Graz, Graz, Austria; Division of Medicine, National Amyloidosis Centre, University College London, Royal Free Hospital, London, UK; Cardiovascular Division, Brigham and Women's Hospital, Harvard Medical School, Boston, MA, USA; Institute of Molecular and Translational Therapeutic Strategies, Hannover Medical School, Carl-Neuberg-Str. 1, Hannover 30625, Germany; Center for Translational Regenerative Medicine, Hannover Medical School, Carl-Neuberg-Str. 1, Hannover 30625, Germany

**Keywords:** Heart failure with preserved ejection fraction, Left ventricular hypertrophy, Cardiac remodelling, Myocardial fibrosis

## Abstract

Heart failure with preserved ejection fraction (HFpEF) accounts for nearly half of all heart failure cases and is characterized by phenotypical heterogeneity with a high prevalence of multiple, often overlapping cardiometabolic disorders. Comorbidities such as hypertension, obesity, or diabetes are present in many HFpEF patients and are hypothesized to contribute to adverse cardiac remodelling and myocardial fibrosis through a variety of haemodynamic and metabolic impairments, with nearly half of all HFpEF patients exhibiting left ventricular (LV) hypertrophy or concentric remodelling. Myocardial fibrosis and its surrogate changes in LV structure and geometry lead to functional impairments such as increased diastolic stiffness and elevated filling pressures and are associated with reduced exercise tolerance and poor prognosis in patients with HFpEF. Despite recent therapeutic progress, there are currently no evidence-based therapies mechanistically focusing solely on myocardial fibrosis and LV hypertrophy in HFpEF. Recognizing myocardial fibrosis and LV hypertrophy as key features of the heterogeneous HFpEF pathophysiology may contribute to the development of promising targets for future clinical trials. This review elaborates on the pathophysiological role of fibrotic remodelling and LV hypertrophy in HFpEF, outlines contemporary diagnostic standards, and discusses emerging therapeutic strategies, aiming at directly modulating myocardial fibrosis and hypertrophy in HFpEF.

## Graphical Abstract

**Graphical Abstract:**
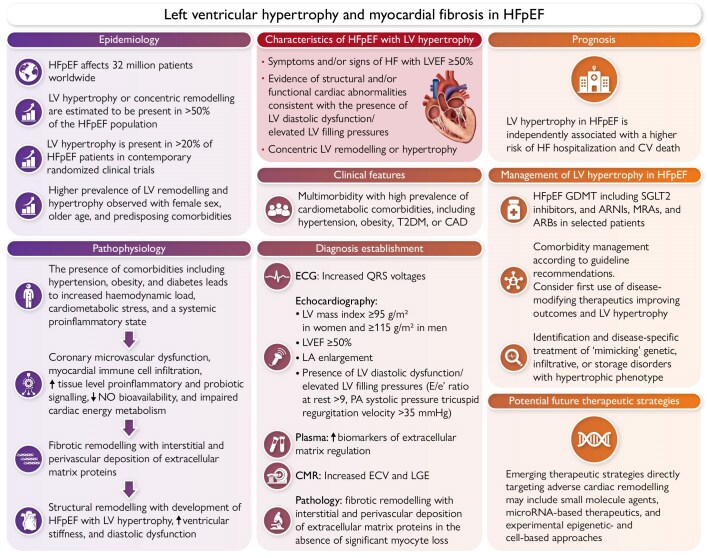
Pathophysiology, phenotypical characteristics, epidemiology, and management strategies of HFpEF with left ventricular hypertrophy. Left ventricular mass index is determined based on LV mass and body surface area. ARB, angiotensin receptor blocker; ARNI, angiotensin receptor neprilysin inhibitor; AV, atrioventricular; CAD, coronary artery disease; CMR, cardiovascular magnetic resonance; CV, cardiovascular; DM, diabetes mellitus; E/e′, early filling velocity on transmitral Doppler/early relaxation; ECV, extracellular volume; GLS, global longitudinal strain; HF, heart failure; HFpEF, heart failure with preserved ejection fraction; LA, left atrium; LV, left ventricle; LGE, late gadolinium enhancement; LVH, left ventricular hypertrophy; PA, pulmonary artery; MRA, mineralocorticoid receptor antagonist; NO, nitric oxide; SGLT2, sodium-glucose cotransporter-2.

## Introduction

Heart failure (HF) with preserved ejection fraction (HFpEF) accounts for nearly half of all HF cases, is increasing in prevalence, and is associated with a substantial morbidity and mortality burden.^1–3^ Patients with HFpEF are characterized by older age and phenotypical heterogeneity, with multiple, often overlapping, comorbidities.^4,5^ Growing evidence suggests pathophysiologically different phenogroups separated by distinct comorbidities and haemodynamic or structural abnormalities, including obesity-related or ischaemic HFpEF.^5–7^ While pathophysiologic mechanisms are complex and may vary by phenotype, the presence of hypertension, diabetes, and obesity is hypothesized to be related to a variety of haemodynamic and cardiometabolic impairments, including pressure or volume overload, systemic inflammation, coronary microvascular dysfunction, capillary rarefaction, reduced nitric oxide bioavailability, and mitochondrial dysfunction, leading to adverse cardiac remodelling and myocardial fibrosis.^8–10^ The resulting structural and functional changes include left ventricular (LV) hypertrophy (LVH) or concentric remodelling, increased myocardial stiffness, and elevated diastolic filling pressures, which represent hallmarks of HFpEF.^11,12^ Recent evidence from contemporary randomized clinical trials suggests that more than half of HFpEF patients exhibit concentric remodelling or LVH, which is associated with an increased risk of HF hospitalizations and cardiovascular (CV) death.^12–14^ Despite the high prevalence of hypertrophic HFpEF and recent therapeutic progress, there are currently no specific therapies mechanistically focusing solely on myocardial fibrosis and LVH. Recognizing myocardial fibrosis and LVH as key features of the heterogeneous pathophysiology of the HFpEF syndrome may contribute to the development of promising targets.^5,15^ This review elaborates on the pathophysiological role of adverse cardiac remodelling and LVH in HFpEF, outlines diagnostic approaches, and discusses emerging therapeutic strategies aiming at directly modulating myocardial fibrosis in hypertrophic HFpEF.

## Definition of left ventricular hypertrophy in heart failure with preserved ejection fraction

While the diagnostic criteria classifying HF subtypes have historically evolved using varying ranges of ejection fraction, contemporary guidelines define HFpEF as symptoms and/or signs of HF with LV ejection fraction (LVEF) ≥ 50% and evidence of structural and/or functional cardiac abnormalities consistent with the presence of LV diastolic dysfunction/elevated LV filling pressures, including increased natriuretic peptide levels and non-invasive and invasive haemodynamic measurements.^16–18^ Left ventricular hypertrophy in HFpEF is characterized by increased LV mass and is frequently accompanied by heterogeneous underlying conditions, including volume or pressure overload, cardiometabolic stress, or genetic disorders, contributing to the development of adverse cardiac remodelling through a complex interplay of pathophysiological mechanisms (*Graphical Abstract*). Although all remodelling patterns leading to the development of LVH involve extracellular matrix (ECM) changes and cardiac fibrosis, LV geometry may vary depending on predominant underlying conditions.^19^ As such, the most common remodelling patterns in HFpEF with LVH include concentric remodelling with increased wall thickness and concentric hypertrophy, whereas eccentric hypertrophy typically occurs more frequently in patients with decreasing LVEF and LV dilation.^20^ Despite sharing common features of LVH in HFpEF, in a considerable subset of patients, specific aetiologies including valvular heart disease and diseases of the myocardium mediated by genetic, or infiltrative disorders, including hypertrophic cardiomyopathy (HCM) and cardiac amyloidosis, with distinct pathophysiology and unique phenotypes may be present.

## Epidemiology of left ventricular structural remodelling and myocardial fibrosis in heart failure with preserved ejection fraction

Although the prevalence may vary depending on patient demographics and comorbidity burden, LVH and concentric remodelling together are estimated to affect 42%–77% of the HFpEF population. In the echocardiography substudies of the I-PRESERVE (Irbesartan in Heart Failure With Preserved Ejection Fraction) and PARAGON-HF (Prospective Comparison of ARNI With ARB Global Outcomes in HF With Preserved Ejection Fraction) trials of patients with symptomatic HF with LVEF ≥ 45%, LV structural remodelling (including LV concentric remodelling and hypertrophy) was prevalent in approximately half of the patients (54%), with LV concentric remodelling [relative wall thickness (RWT) < 0.42] present in 25%–33% and LVH (LV mass index ≥ 95 g/m^2^ in women and ≥115 g/m^2^ in men, respectively) in 21%–29%.^12,13^ Conversely, a higher prevalence of structural abnormalities (77%) with concentric LV remodelling being present in 34% and LVH in 52% was observed in the echocardiography substudy of the TOPCAT (Treatment of Preserved Cardiac Function Heart Failure with an Aldosterone Antagonist) trial of patients with symptomatic HF with LVEF ≥ 45%.^21^ Compared with randomized clinical trials, in community-based cohorts and hospital-based registries of HFpEF, a consistent prevalence of concentric LV remodelling was observed (20%–28%), whereas higher proportions of patients had LVH (42%–75%).^22–26^ Although concentric LVH and remodelling are considered predominant patterns of structural LV abnormalities in HFpEF, of the 21% of patients with LVH in the PARAGON-HF echocardiography substudy, a concentric LVH pattern was observed in 11%, while an eccentric LVH pattern was present in 10%.^13^ Comparable rates of eccentric hypertrophy patterns were observed in the Northwestern HFpEF Registry (12%) and participants with HFpEF in the Olmsted County cohort (16%).^22,25^

Of note, LV geometric patterns can further be affected by demographic factors such as age, sex, race, and comorbidities.^27^ As such, age is a fundamental independent determinants of increments in LV mass.^28,29^ Women are subject to a greater and steeper increase of LV mass with aging, resulting in LV remodelling and hypertrophy being present to a greater degree in women, compared with men.^30,31^ Moreover, LV remodelling and hypertrophy are reportedly more common in individuals with key predisposing comorbidities, with the strongest association observed with hypertension, followed by diabetes, obesity, and coronary artery disease (CAD).^12,32^While race- and ethnic-specific data on LV geometry patterns in HFpEF patients remain limited, in the MESA (Multi-Ethnic Study of Atherosclerosis) cohort, the prevalence of LVH was higher in Hispanics and non-Hispanic Blacks without an established diagnosis of HFpEF compared with non-Hispanics, irrespective of age, sex, comorbidities, socioeconomic, and behavioural factors.^33^ Likewise, eccentric LVH was reported to be more prevalent than concentric patterns in response to hypertension among Black individuals without HF, compared with Whites, in the Bogalusa Heart Study.^34^

Consistent with the observed echocardiographic prevalence of concentric LV remodelling and hypertrophy, myocardial fibrosis, which represents a key feature of hypertrophic HFpEF, was shown to be present in LV structural remodelling, affecting more than 41% of patients with HFpEF as determined by extracellular volume (ECV) and post-contrast T1 mapping cardiovascular magnetic resonance (CMR) measures in population based-cohort studies performed at the Medical University of Vienna and the University of Pittsburgh School of Medicine.^8,35^ Of note, histologically detected fibrosis from LV biopsies was demonstrated to be correlated with CMR surrogates of myocardial fibrosis.^35^ While data from myocardial tissue analyses in patients with HFpEF remain limited, a large prospective endomyocardial biopsy study conducted at the Johns Hopkins HFpEF Clinic further revealed the high prevalence of myocardial fibrosis and myocyte hypertrophy in HFpEF (93% and 88%, respectively), although these features often present in mild severity (66% with fibrosis and 45% with myocyte hypertrophy).^36^ Nevertheless, hypertrophic phenotypes exhibiting moderate or severe myocardial fibrosis and myocyte hypertrophy were present in 34% and 43%.^36^

Importantly, it should be acknowledged that the estimated prevalence of cardiac amyloidosis, with ATTR amyloidosis accounting for the majority of the cases, is ∼15% in patients with HFpEF based on tissue analyses from endomyocardial biopsy, and nearly one-half of the estimated cases of cardiac amyloidosis remained undiagnosed.^36–38^ Moreover, HCM is anticipated to have a considerable prevalence of 2–5/1000 of the general population, accounting for 2%–3% of HF cases.^39^

## Pathophysiology of left ventricular hypertrophy in heart failure with preserved ejection fraction and other cardiomyopathies with hypertrophic left ventricular geometry

### Left ventricular hypertrophy in heart failure with preserved ejection fraction

**Figure 1:**
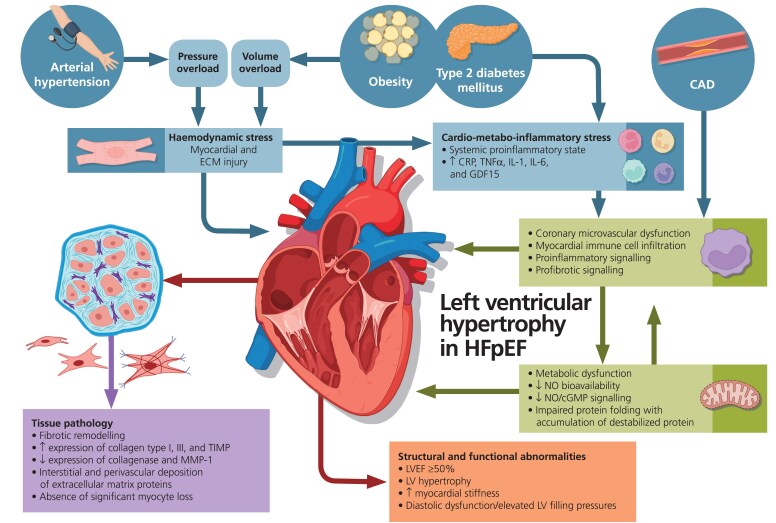
Pathophysiology of left ventricular hypertrophy in HFpEF. CRP, C-reactive protein; GDF15, growth differentiation factor 15; HFpEF, heart failure with preserved ejection fraction; IL-1, interleukin-1; IL-6 interleukin-6; LV, left ventricle; LVEF, left ventricular ejection fraction; MMP-1, metalloproteinase-1; NO, nitric oxide; cGMP, cyclic guanosine monophosphate; TIMP, tissue inhibitor of metalloproteinase, TNFα, tumour necrosis factor alpha

The development of LVH in HFpEF is presumed to be driven by multiple interacting comorbidities, including hypertension, obesity, and diabetes, through mechanisms ranging from increased haemodynamic load, cardiometabolic stress, inflammation, microvascular dysfunction to pathophysiological remodelling, and cardiac fibrosis (*Figure 1*).^40^ Increased haemodynamic load is an important pathomechanism in LVH activating proinflammatory and profibrotic signalling, being predominantly caused by pressure overload due to hypertension, with obesity and diabetes mellitus further contributing to volume overload due to elevated plasma volume, neurohumoral activation, and alterations in sodium handling.^41–46^ In addition to haemodynamic load-related signalling, a broad range of cardiometabolic disorders are further accompanied by a systemic proinflammatory state with elevated levels of C-reactive protein, tumour necrosis factor α (TNFα), interleukin-6 (IL-6), growth differentiation factor 15 (GDF15), interleukin-1 (IL-1) receptor 1, and IL-1 receptor-like 1 resulting in microvascular endothelial dysfunction with myocardial immune cell infiltration, together with impairments in nitric oxide bioavailability, altered nitric oxide/cyclic guanosine monophosphate signalling, and protein folding with accumulation of destabilized proteins.^10,47^ Collectively, changes in the interstitial tissue environment due to immune cell infiltration, triglyceride oversupply, increased cardiometabolic stress, and proinflammatory and profibrotic signalling lead to ECM alterations with elevated expression of collagen Type I, Type III, tissue inhibitor of metalloproteinase, but reduced collagenase and metalloproteinase-1 expression.^9,48,49^ In addition to interstitial cells such as fibroblasts, cardiac remodelling affects all tissue components including myocytes and endothelial cells.^50^ As part of the maladaptive cardiac remodelling process, cardiac fibroblast activation is crucially regulated by the transforming growth factor β (TGF-β) family at least in part through modulation of microRNAs (miR), while it may also be altered by other inflammatory or fibrogenic mediators, mechanical, paracrine, and humoral signals issued from the microenvironment.^51,52^ The progression of fibrotic remodelling with interstitial and perivascular deposition of ECM proteins in the absence of significant myocyte loss ultimately results in increased passive myocardial stiffness, LVH, and diastolic dysfunction, representing hallmarks of the hypertrophic HFpEF phenotype.^12^

Eccentric hypertrophic patterns with dilated chambers occur primarily following volume overload remodelling due to a predominantly matrix-degrading environment with matrix-degrading protease activation and reduction of interstitial collagen.^53–55^ Conversely, concentric remodelling, characterized by enhanced ECM synthesis resulting from neurohumoral activation, induction of mechanosensitive fibrogenic pathways, growth factor signalling, and inflammation, appears to be more common with older age, pressure overload, and metabolic impairments such as diabetes, hyperleptinaemia, myocardial steatosis, and visceral adipose tissue expansion and is the predominant structural pattern observed in HFpEF.^6,48,56–58^ Depending on their comorbidity burden, patients may exhibit features of both hypertrophy patterns, with the LV remodelling phenotype depending on the dominant mediating factor.

Diabetes and obesity are major risk factors for systemic inflammation and metabolic alterations associated with fibrotic expansion of the cardiac ECM in patients at risk of developing and those with established HFpEF.^56,59^ Given the concomitant presence of metabolic, inflammatory, and haemodynamic stress, most HFpEF patients with diabetes or obesity exhibit remodelling patterns with LVH, atrial myopathy, and right ventricular enlargement.^60^ However, compared with non-obese patients, LV remodelling in obese HFpEF patients often shows features of dilation due to the enhanced plasma volume, together with concentric remodelling patterns with an elevated ratio of LV mass to volume.^6^ Although pathomechanisms remain incompletely understood, specific effects of hyperglycaemia on the myocardium include fibroblast activation induced by the accumulation of advanced glycation end products cross-linking ECM proteins, stimulation of TGF-β signalling, resulting in the deposition of structural ECM proteins and matricellular macromolecules.^61^ Additional pathways stimulating fibroblast activation in diabetes and obesity may comprise adipokine and leptin signalling, endothelin-1 signalling, and renin–angiotensin–aldosterone system (RAAS) activation.^61,62^ Moreover, besides promoting epicardial adipose tissue expansion, diabetes and obesity further result in dysregulated lipid metabolism leading to excessive intracardiac triglyceride accumulation, contributing to myocardial steatosis and lipotoxicity.^63,64^

Atrial fibrillation (AF) is characterized by structural, electrical, and functional atrial remodelling. Accordingly, atrial fibrosis is a crucial feature of structural remodelling in AF and is associated with its development and progression.^65^ Atrial structural remodelling and fibrosis may develop due to conditions associated with atrial overload and wall stretch, such as hypertension, valvular heart disease, HF, or aging-related changes.^65^ If present, ventricular fibrosis is more severe in AF patients, particularly in those with permanent or persistent AF, compared with those with sinus rhythm or paroxysmal AF, although detectable ventricular fibrosis develops over an extended period providing it is not aggravated by comorbidities.^66,67^ However, while evidence from patients with HFpEF remains limited, suggested key mechanisms of atrial and ventricular fibrosis in patients with AF include haemodynamic impairments, mechanical stress, neurohormonal changes, growth factors, and proinflammatory cytokine signalling.^65^

Aging-related cardiac remodelling is characterized by bi-ventricular and bi-atrial accumulation of ECM proteins with concentric LV remodelling.^48,68^ With older age, production and cross-linking of fibrillar collagen tend to further increase, while collagen degradation may be attenuated.^69^ The process is driven by the expansion of epicardial-derived fibroblasts infiltrating the cardiac interstitium, promoting reactive oxygen species, neurohumoral activation, TGF-β, and proinflammatory cytokine and chemokine signalling.^70^ Moreover, fibroblasts in the aging heart are suggested to be characterized by a proinflammatory phenotype with enhanced osteogenic gene expression, impaired endothelial angiogenic activation, and enhanced inflammation.^71,72^ Similarly, reparative fibroblast functions may be further limited by aging-related impairments in responses to fibrogenic growth factors.^73^ Additional mechanisms involved in senescent hearts include disruption of intracellular calcium homoeostasis, cardiomyocyte apoptosis, and impaired mitochondrial function.^74^

### Phenocopies of heart failure with preserved ejection fraction with left ventricular hypertrophy and distinct pathophysiology

Although LVH due to aortic stenosis shares similar features of pressure overload-induced fibrotic remodelling with comparable geometric and functional LV changes observed in HFpEF patients with uncontrolled hypertension, cardiomyopathy associated with aortic stenosis should be considered a separate entity due to its distinct aetiology.^75^ Hypertrophic cardiomyopathy is a relatively common predominantly inherited cardiomyopathy often caused by sarcomere gene mutations.^76^ While HCM likely represents a considerable cause of LVH in patients with HFpEF, its phenotype is complex with features of asymmetric LVH disproportionate to the degree of LV loading conditions in the absence of other cardiac, metabolic, or systemic disease linked to a comparable increase in LV mass.^39^ Although LVH mainly affects the basal and mid-interventricular septum, other segments, including multiple locations, may be involved.^11^ Mechanistically, sarcomere mutations lead to myocyte hypertrophy and disarray, driven by disrupted energy balance and increased energy consumption to maintain hyperdynamic sarcomere tension.^77^ As a result of sustained depletion of myocyte energy, together with impairments related to ischaemic stress due to microvascular dysfunction, a gradual myocyte loss with replacement fibrosis occurs, further contributing to adverse LV remodelling, systolic dysfunction, and the development of HF.^39^ Despite the absence of an increase in haemodynamic load, according to data from histological analyses of explanted hearts from patients with end-stage HCM, more than one-third of the LV myocardium may be replaced by fibrosis during the disease trajectory, suggesting the involvement of profibrotic mediators.^78^ Cardiac amyloidosis, despite sharing the feature of increased wall thickness, is a pathophysiologically distinct and progressive infiltrative disease.^79^ In over 98% of diagnosed cases, it arises from either monoclonal immunoglobulin light chains (AL) originating in the bone marrow or transthyretin (TTR), a liver-produced transporter protein.^79^ Transthyretin (ATTR) amyloidosis includes hereditary (ATTRm) and wild-type (ATTRwt) forms, with ATTRwt amyloidosis being particularly prevalent with older age while affecting a substantial proportion of patients with HFpEF.^80^ In contrast to hypertrophic HFpEF, interstitial fibrosis is not an integral feature of cardiac amyloidosis, in which organ damage and wall thickening are determined by the persistent deposition of misfolded amyloid fibrils in the extracellular space, leading to acute proteotoxic effects and pathologic remodelling with the development of a concentric biventricular hypertrophy.^37^ Fabry disease is a rare hereditary disorder caused by pathogenic variants in the GLA gene, resulting in deficient or absent alpha-galactosidase A (α-Gal A) activity and subsequent lysosomal accumulation of globotriaosylceramide (Gb3), affecting the heart, vessels, kidney, and peripheral nervous system.^81,82^ While Fabry disease always affects male carriers based on its X-linked inheritance, females may develop organ involvement later in life potentially reaching similar severity due to the lyonization phenomena with X-chromosome inactivation.^83^ Depending on the genetic variant, α-Gal A activity and Gb3 accumulation may vary with distinct onset age, organ manifestations, and symptoms.^84^ In the myocardium, the accumulation of Gb3 leads to biochemical and functional impairment in myocytes with activation of hypertrophy pathways leading to progressive wall thickening, microvascular dysfunction with ischaemia, myocyte loss, and replacement fibrosis, resulting in LV dysfunction and development of ventricular arrhythmias and conduction system impairments.^85^

## Diagnostic evaluation of left ventricular hypertrophy in heart failure with preserved ejection fraction

### Multimodality imaging and classification of left ventricular structure

Concentric LV remodelling and hypertrophy are hallmarks of HFpEF. Based on the recommendations of the American Society of Echocardiography and the European Association of Cardiovascular Imaging, LV geometry is classified by LV mass index (LV mass/body surface area) and RWT calculated by the formula 2 × posterior wall thickness/LV internal diameter at end-diastole.^20^ While LVH is defined as an increase in LV mass index ≥ 95 g/m^2^ in women and ≥115 g/m^2^ in men, respectively, RWT further allows to categorize increased LV mass as concentric (RWT > 0.42) or eccentric (RWT ≤ 0.42) LVH.^20^ Normal LV mass with increased RWT indicates the presence of concentric remodelling, whereas patients with normal RWT and LV mass are considered to have normal LV geometry.^20^ Although impairments in LVEF may not be present, concentric remodelling and LVH in HFpEF may be accompanied by impairments in longitudinal strain and myocardial shortening.^86^ In addition to echocardiography, CMR can complement the assessment of LV geometry with detailed tissue characterization and quantification of myocardial fibrosis and steatosis by ECV assessment and T1 mapping in hypertrophic HFpEF.^35,87^ Moreover, detecting early changes in myocardial composition with increased ECV via CMR among patients with arterial hypertension, diabetes, or obesity may help to identify individuals at risk for developing HFpEF.^11^

### Biomarkers of myocardial fibrosis and left ventricular hypertrophy

In addition to multimodality imaging, biomarkers may provide additional value during the diagnosis and management of LVH and fibrosis in HFpEF.^88–90^ Natriuretic peptides are released by cardiomyocytes in response to myocardial stress and serve as widely used diagnostic and prognostic markers of HF across the spectrum of LVEF.^91^ Although elevated circulating N-terminal pro-B-type natriuretic peptide (NT-proBNP) levels are associated with multiple pathways related to fibrosis and inflammation in patients with HFpEF, NT-proBNP levels do not specifically predict myocardial fibrosis and LVH and may vary depending on sex, race, renal function and the presence of obesity.^92–95^ While changes in ECM homoeostasis mainly occur within the myocardium, the synthesis and degradation of ECM proteins, including collagen, are reflected by circulating biomarkers.^96,97^ Propeptides including N-terminal propeptide of collagen I (PINP) and N-terminal propeptide of collagen III (PIIINP) are released during the synthesis of collagen and have been shown to correlate with myocardial depositions of collagen Types I and III, respectively.^98,99^ Markers of collagen turnover and degradation tend to be decreased during profibrotic conditions, including carboxyl-terminal telopeptide of collagen Type I (CITP) and matrix metalloproteinases, together GDF15, a distant member of the TGF-β superfamily, correlates with oxidative stress and inflammation in HF and has been shown to be associated with LVH, diastolic dysfunction, and CMR-assessed myocardial fibrosis.^100–102^ ST2 (sST2) is part of the IL-1 receptors family associated with LVH promoting the activation of fibroblasts and reflecting proinflammatory and profibrotic states.^103^ Likewise, elevated concentrations of galectin-3 (GAL-3), a β-galactoside-binding member of the lectin family involved in the proliferation and differentiation of fibroblasts and macrophages, have been shown to be associated with LVH in myocardial fibrosis.^95,104^ In addition, circulating plasma levels of miR, such as miR-132, may further reflect TGF-β family-mediated cardiac fibroblast activation and have been shown to be elevated and associated with increased disease severity as determined by New York Heart Association (NYHA) functional class in patients with HF.^105^

### Differential diagnoses and phenocopies of heart failure with preserved ejection fraction with left ventricular hypertrophy

**Figure 2:**
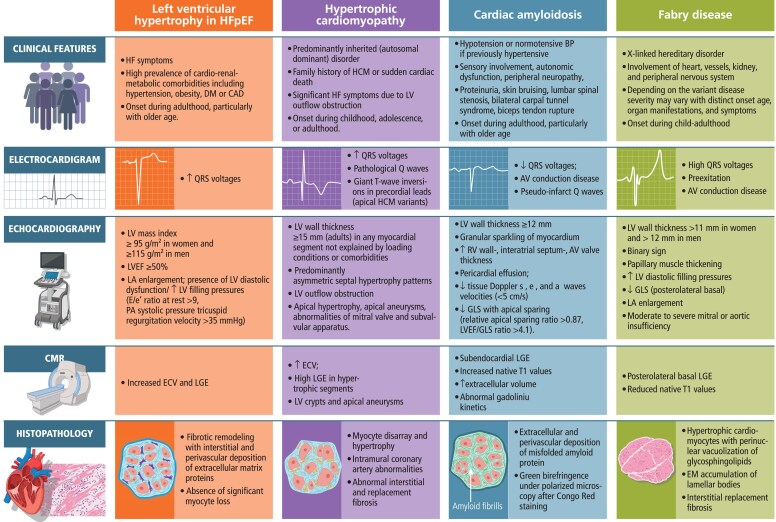
Differential characteristics and diagnosis of HFpEF with left ventricular hypertrophy and other cardiomyopathies with hypertrophic phenotypes. Left ventricular mass index is determined based LV mass and body surface area. AV, atrioventricular; BP, blood pressure; CAD, coronary artery disease; CMR, cardiovascular magnetic resonance; DM, diabetes mellitus; E/e′, early filling velocity on transmitral Doppler/early relaxation; ECV, extracellular volume; EM, extracellular matrix; GLS, global longitudinal strain; HCM, hypertrophic cardiomyopathy; HFpEF, heart failure with preserved ejection fraction; LA, left atrium; LV, left ventricle; LGE, late gadolinium enhancement; LVH, left ventricular hypertrophy; PA, pulmonary artery

Due to the pathophysiological heterogeneity underlying LVH in HFpEF, it is important to consider specific conditions with overlapping morphological and functional features requiring disease-directed diagnostic and therapeutic strategies, including HCM, infiltrative cardiomyopathies (e.g. amyloidosis, haemochromatosis, or sarcoidosis), storage disorders (e.g. Fabry disease), or valvular heart disease (e.g. aortic stenosis). Suspected specific aetiologies require further comprehensive evaluation based on medical history, physical examination, electrocardiogram, targeted multimodality imaging including CMR, biomarkers, genetic testing, invasive haemodynamic assessment, and endomyocardial biopsy (*Figure 2*).^11,49^ Although CMR late gadolinium enhancement can provide important diagnostic information on specific cardiomyopathies, diminishing the need to perform an endomyocardial biopsy, tissue diagnosis is sometimes needed to definitely establish the diagnosis of infiltrative or storage disorders.^106,107^ Because patients with arterial hypertension, which causes the majority of cases with LVH, are at increased risk of developing CAD, the exclusion of myocardial ischaemia may be additionally considered. Although LVH may be present in patients with athlete's heart, this phenotype is characterized by balanced hypertrophy of myocytes and collagen without adverse remodelling, normal diastolic elasticity, and filling pressures.^11^

## Prognosis

The presence of LVH in HFpEF as determined by increased LV mass index has been shown to be independently associated with a higher risk of HF hospitalization and CV death, and incident HF hospitalizations in clinical trials, including I-PRESERVE, TOPCAT, and PARAGON-HF, and prospective hospital-based cohort studies.^12–14,25^ Specifically, for the composite of HF hospitalizations and CV death, LVH was associated with a hazard ratio (HR) of 1.86 [95% confidence interval (CI) 1.40–2.46) in I-PRESERVE, 1.58 (95% CI 1.22–2.05) in TOPCAT, and an adjusted HR of 1.40 (95% CI 1.16–1.70) for categorical analyses and 1.08 (1.04–1.13) per 10 g/m^2^ increase in LV mass index, respectively, in PARAGON-HF.^12–14^ Concordant-independent associations were similarly observed for E/e′ ratio and pulmonary artery systolic pressure. Given the consistent independent associations of LV mass index with HF hospitalization and CV death in clinical trials and cohort studies in patients with HFpEF and its routine assessment in clinical echocardiography practice, its use supports the identification of individuals at increased hypertrophy-related risk. Likewise, myocardial fibrosis quantified by CMR measures including ECV and T1 mapping has been shown to be independently associated with elevated natriuretic peptides and heightened risk for the composites of all-cause death or HF hospitalizations (adjusted HR 1.75 per 5% increase in ECV, 95% CI 1.25–2.45) in a prospective cohort study.^8^ As shown with imaging markers of LVH and fibrosis, biomarkers of ECM haemostasis, including sST2, TIMP-1, and GAL-3, were independently associated with an increased risk of adverse HF events and CV mortality in patients with HFpEF and thus may provide additional prognostic value over other conventional biomarkers such as NT-proBNP.^88–90^

## Contemporary and future treatment strategies for left ventricular hypertrophy in heart failure with preserved ejection fraction

### Antihypertrophic and antifibrotic properties of contemporary management strategies for heart failure with preserved ejection fraction

**Figure 3:**
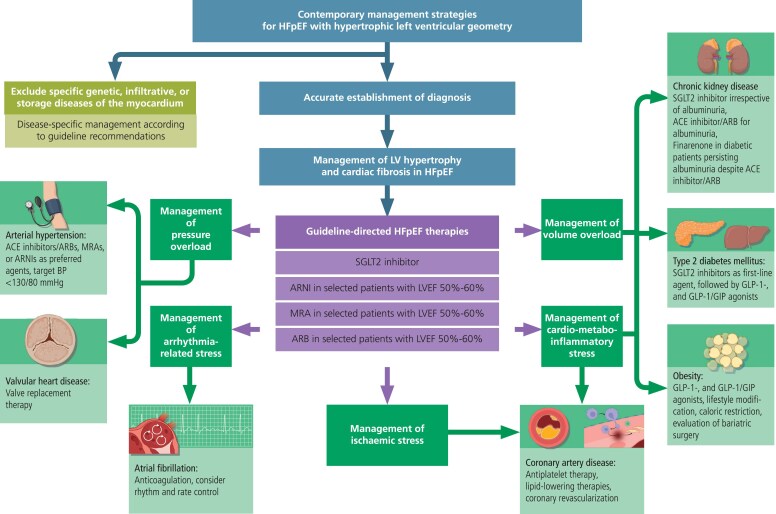
Management of left ventricular hypertrophy in HFpEF. ARB, angiotensin receptor blocker; ARNI, angiotensin receptor neprilysin inhibitor; BP, blood pressure; GLP-1RA, glucagon-like peptide 1 receptor agonist; GIP, glucose-dependent insulinotropic polypeptide; HFpEF, heart failure with preserved ejection fraction; LVEF, left ventricular ejection fraction; MRA, mineralocorticoid receptor antagonist; SGLT2, sodium-glucose cotransporter-2

Although contemporary evidence-based medical therapies for the management of HFpEF and related comorbidities are not solely targeting LV remodelling and myocardial fibrosis, many agents may also attenuate LVH and myocardial fibrosis (*Figure 3*).^16,108^ There is evidence from randomized trials of HF that agents inhibiting the RAAS may attenuate LVH and myocardial fibrosis.^109–111^ In meta-analyses of patients with HFpEF and diastolic dysfunction, the use of steroidal mineralocorticoid receptor antagonists (MRAs) including spironolactone or eplerenone has been shown to reduce LV mass and markers of cardiac fibrosis.^112,113^ Compared with commonly used steroidal MRAs, emerging non-steroidal MRAs, such as finerenone, which has been shown to reduce total worsening HF events and CV mortality in the FINEARTS-HF trial of patients with symptomatic HF and LVEF ≥ 40, have a higher mineralocorticoid receptor affinity and selectivity, with preclinical data suggesting greater antifibrotic, anti-inflammatory, and antihypertrophic effects.^114–116^ Moreover, angiotensin receptor neprilysin inhibitor (ARNI) treatment favourably altered makers of ECM homoeostasis in the PARAGON-HF (Prospective Comparison of ARNI With ARB Global Outcomes in HF With Preserved Ejection Fraction) trial of patients with symptomatic HF and LVEF ≥ 45%.^88^ In addition, while data on LV mass from HFpEF patients remain limited, ARNI use was shown to reduce LV mass in patients with hypertensive heart disease.^117^ Likewise, angiotensin-converting enzyme inhibitors and angiotensin receptor blockers (ARB) were demonstrated to mediate regression of myocardial fibrosis and ventricular stiffness in patients with hypertensive heart disease.^118,119^ The antihypertrophic effects observed with RAAS inhibition are thought to be in part mediated by haemodynamic improvements through blood pressure reduction in addition to direct effects on cardiomyocytes, cardiac fibroblasts, endothelial cells, and vascular smooth muscle cells.^120–122^ As such, the degree of blood pressure reduction does not correlate with the magnitude of LVH regression, whereas blockade or inhibition of both Angiotensin II and mineralocorticoid receptors was shown to have additive effects in patients with hypertensive heart disease.^123^ While the effects of blood pressure lowering on ventricular structure may be attenuated in women and obese individuals with hypertensive heart disease, there is no evidence of a differential treatment response to RAAS inhibition on cardiac structure and function across clinical HFpEF phenogroups.^113,124^ Attenuated responses in women and among obese subjects suggest that structure–function changes may be less reversible in these groups. In addition to their benefits on clinical events and functional health-related quality of life, sodium-glucose cotransporter-2 (SGLT2) inhibitor treatment may also reduce LVH.^125,126^ In the EMPA-HEART (Effects of Empagliflozin on Cardiac Structure in Patients with Type 2 Diabetes) CardioLink-6 trial of patients with Type 2 diabetes at high CV risk, SGLT2 inhibitor treatment was associated with a significant reduction in LV mass.^127^ While exact mechanisms mediating the clinical benefits remain incompletely understood, it is possible that improvements in clinical outcomes may be in parts related to antifibrotic effects. Indeed, in the EMPA-REG OUTCOME (Empagliflozin, Cardiovascular Outcomes, and Mortality in Type 2 Diabetes trial) trial of patients with Type 2 diabetes at high CV risk, SGLT2 inhibitor treatment showed direct effects on human cardiac myofibroblast phenotypes and function by attenuation of myofibroblast activity and cell-mediated collagen remodelling, with an *ex vivo* single-cell sequencing study of human cardiac tissue from patients with HF further indicating cell-type-specific antifibrotic expression patterns in fibroblasts with SGLT2 inhibitor use.^128,129^ Emerging antiobesity drugs such as combined glucagon-like peptide-1/glucose-dependent insulinotropic polypeptide (GLP-1/GIP) receptor agonists cause considerable weight loss and were shown to reduce LV mass and RWT in addition to their benefits on the risk of CV death, worsening HF, and improvements in health status among patients with obesity-related HFpEF.^130,131^ While evidence from clinical trials remains limited, experimental data indicate that combined GLP-1/GIP agonists may further limit diabetes-related cardiac fibrosis.^132^ Likewise, in support of the benefits on functional status, weight loss through bariatric surgery is associated with significant reductions in features of LVH in patients with hypertrophic HFpEF.^133^ Conversely, the GLP-1RA semaglutide did not significantly affect LV dimensions or mass in the STEP-HFpEF (Semaglutide Treatment Effect in People with Obesity and HFpEF) programme echocardiography substudy, which may be due to the comparatively lower extent of body weight reduction with semaglutide compared with combined GLP-1/GIP agonists or bariatric surgery.^134^ Because CAD is present in a relevant number of patients with hypertrophic HFpEF, coronary revascularization to reduce ischaemia may improve LV structural remodelling and prognosis.^135,136^ Important additional considerations for the management of comorbidities to attenuate adverse cardiac remodelling and fibrosis in patients with hypertrophic HFpEF include treatment of AF, valvular heart disease, sleep apnoea, and chronic kidney disease (CKD).^65,137–139^

### Device-based strategies

In addition to pharmacological therapies, selected HFpEF patients with LVH and resistant hypertension may benefit from interventional or device-based strategies. Evidence from randomized trials of patients with resistant hypertension and LVH, in parts fulfilling HFpEF criteria based on the definition from the European Society of Cardiology, and data from registries of HFpEF patients with resistant hypertension suggest that renal sympathetic denervation (RDN) may reduce features of LVH and improve diastolic function.^140–143^ While the benefits on LV structure in patients with hypertensive heart disease were in parts observed irrespective of blood pressure changes, experimental data from rodent models suggest that potential improvements with RDN potentially being mediated by to attenuated renal fibrosis and inflammation via reduced expression of proinflammatory NOD-like receptor family pyrin domain containing 3 (NLRP3)/interleukin-1β signalling and profibrotic mediators.^144,145^ Moreover, baroreflex activation therapy (BAT) may mitigate pressure overload by limiting sympathetic nervous system activation and enhancing parasympathetic nervous system effects through implantable programmable pulse generator devices. Evidence from patients with HFpEF and resistant hypertension from a small prospective observational study conducted at the University Medical Centre Göttingen and a substudy of patients with resistant hypertension enrolled in the DEBuT-HT (Device Based Therapy in Hypertension Extension Trial) and two US feasibility trials indicate that BAT is associated with improved filling pressures, features of LVH, and left atrial size beyond reducing systolic blood pressure.^146,147^ Although RDN and BAT demonstrated promising reverse remodelling effects on LVH in HFpEF, their effects on symptoms and clinical outcomes in patients with hypertrophic HFpEF and resistant hypertension remain to be further examined.

### Emerging therapeutic strategies for left ventricular hypertrophy in heart failure with preserved ejection fraction directly targeting adverse cardiac remodelling

Given the high prevalence and the important prognostic implications of LV remodelling and hypertrophy in HFpEF, with myocardial fibrosis and exaggerated ECM production presenting key features of adverse cardiac structural remodelling, phenotype-specific targeting of LVH with antifibrotic therapeutics may address significant unmet needs. Emerging agents in preclinical and clinical development aiming at promising novel molecular targets, including cellular therapies, epigenetic modifications, and regulatory RNAs, may introduce new strategies to counteract adverse cardiac remodelling and fibrosis in hypertrophic HFpEF (*Table 1*).

**Table 1:** Contemporary and future treatment strategies modulating myocardial hypertrophy and fibrosis in heart failure with preserved ejection fraction

Treatment strategy	Mechanism of action	Effect on LV hypertrophy and cardiac fibrosis	Study	Level of evidence	Reference
**Contemporary HFpEF management strategies**					
SGLT2 inhibitors	Reduction of haemodynamic load, improvement of cardiac cellular metabolism with promotion of autophagy, and restoration of mitochondrial health	Reduction in LV mass	Patients with T2DM at high cardiovascular risk	Phase 3 RCT	^127,128^
		Attenuation of myofibroblast activity and cell-mediated collagen remodelling	*Ex vivo* study of human cardiac tissue from patients with HF	Observational case survey	^129^
MRA	Reduction of haemodynamic load, direct effects on cardiomyocytes, cardiac fibroblasts, endothelial cells, and vascular smooth muscle cells	Reduction in LV mass and markers of cardiac fibrosis	Patients with symptomatic HFpEF	Meta-analyses of Phase 3 RCTs	^112,113^
ARNIs	Reduction of pressure and volume load, direct effects on cardiomyocytes, cardiac fibroblasts, endothelial cells, and vascular smooth muscle cells	Favourable alteration of ECM makers	Patients with symptomatic HFpEF	Phase 3 RCT	^88^
		Reduction in LV mass	Patients with hypertensive heart disease	Phase 2 RCT	^117^
GLP-1RAs	Weight loss, reduction in systemic inflammation, attenuated haemodynamic load	No significant effect on LV dimensions or mass	Patient-level pooled analysis of 2 trials of obesity-related HFpEF with and without T2DM	2 Phase 2RCTs	^134^
GLP-1/GIP agonists	Weight loss (more pronounced compared with GLP1-RA), reduction in systemic inflammation, attenuated haemodynamic load	Reduction in LV mass and RWT	Patients with obesity-related HFpEF	Phase 3 RCT	^131^
		Limitation of diabetes-related cardiac fibrosis	Human cardiac AC16 cell line	Experimental (*in vitro*)	^132^
**Emerging therapeutic strategies currently under investigation**					
Small molecule TGF-β inhibitors	Modulating maladaptive fibroblast differentiation	Reduction in myocardial fibrosis and NT-proBNP levels	Patients with HFpEF and CMR evidence of myocardial fibrosis	Phase 2 RCT	^148^
Antisense oligonucleotide miR-132 inhibitors	Modulation of signalling pathways leading to adverse cardiac remodelling and fibrosis by regulating cardiomyocyte growth, autophagy, calcium handling, and fibrosis pathways	Effect on cardiac function and safety currently under investigation	Patients with LVEF ≤ 45% after myocardial infarction	Ongoing Phase 2 RCT	^149^
		Safe, dose-dependent, sustained miR-132 reduction in plasma	Patients with chronic HF with LVEF between ≥30% and <50%	Phase 1b RCT	^150^
		Improvement in cardiac function and reduced cardiac dilatation	Pressure overload mouse models, post-MI pig models of HF	Preclinical	^151^
Antisense oligonucleotide miR-21 inhibitors	Regulation of cardiac fibroblast survival via ERK-MAPK signalling	Enhanced cellular viability leading to improved cardiac function, evidenced by shorter relaxation time	*Ex vivo* model of living explanted human myocardium from patients with HF	Observational case survey	^152^
		Reduction of cardiac fibrosis	Pressure overload mouse models, post-MI pig models of HF	Preclinical	^153, 154^
**Potential novel molecular targets**					
Endogenously reprogrammed T cells and adaptive transfer of exogenously derived CAR-T cells to target FAP	Specific targeting of cardiac fibroblast activation	Reduction in cardiac fibrosis and improvements in systolic function	Pressure overload mouse models	Preclinical	^155,156^
Adoptive transfer of regulatory T cells	Limitation of inflammatory signalling, myocardial expression of inflammatory cytokines, and cardiac immune cell infiltration	Attenuated cardiac remodelling, fibrosis, and function	Pressure overload mouse models	Preclinical	^157,158^
Genetic and antibody-based ablation of CCR2+ macrophages	Inhibition of cardiac fibroblast activation through CCR2+ macrophages derived profibrotic inflammatory cytokines	Attenuated cardiac remodelling, fibrosis, and function	Pressure overload mouse models	Preclinical	^159^
Antibody-based blockade and deletion of IL-1 receptors in cardiac fibroblast	Reduced secretion of fibroblast-derived inflammatory cytokines	Reduced markers of cardiac fibrosis	Pressure overload mouse models	Preclinical	^160,161^
Secretome inhibition in proinflammatory macrophages	Reductions in macrophage-derived IL-6 and TNFα secretion limiting cardiac fibroblast activation	Limited interstitial fibrosis and cardiac dysfunction	Pressure overload mouse models	Preclinical	^162^
Inhibition of fibrogenic cross-linking enzymes	Blockade of tissue transglutaminase, leading to ECM modulation by suppression of fibrosis-associated gene expression	Improved diastolic function, reduced cardiomyocyte hypertrophy and fibrosis	Pressure overload mouse models	Preclinical	^163^
Epigenetic modulation of fibroblast activation	Inhibition of histone deacetylases, histone acetyltransferases, bromodomain extraterminal protein, and lysine demethylases to reduce fibroblast activation	Attenuated cardiac hypertrophy and fibrosis	Pressure overload mouse models	Preclinical	^164–166^

ARB, angiotensin receptor blocker; ARNI, angiotensin receptor neprilysin inhibitor; BP, blood pressure; CAR, chimeric antigen receptor; CCR2, C-C Motif Chemokine Receptor 2; ECM, extracellular matrix; ERK-MAPK, extracellular signal-regulated mitogen-activated protein kinase; FAP, fibroblast activating protein; IL-1, interleukin-1; IL-6, interleukin-6; GLP-1RA, glucagon-like peptide 1 receptor agonist; GIP, glucose-dependent insulinotropic polypeptide; HF, heart failure; HFpEF, heart failure with preserved ejection fraction; LVEF, left ventricular ejection fraction; MI, myocardial infarction; miR-21, microRNA-21; miR-132, microRNA-132; MRA, mineralocorticoid receptor antagonist; NT-proBNP, N-terminal pro-B-type natriuretic peptide; RWT, relative wall thickness; SGLT2, sodium-glucose cotransporter-2; T2DM, Type 2 diabetes mellitus; TGF-β, transforming growth factor β; TNFα, tumour necrosis factor α.

### Small molecule antifibrotic agents

Evidence from experimental and clinical studies indicates that inhibiting TGF-β signalling, a key modulator of maladaptive fibroblast differentiation in cardiac remodelling, may limit cardiac fibrosis.^167^ Pirfenidone is an oral small molecule antifibrotic agent inhibiting TGF-β secretion and reducing cardiac fibroblast synthesis, which is approved for the management of idiopathic pulmonary fibrosis.^168^ In the Phase 2 PIROUETTE (Pirfenidone in Patients with Heart Failure and Preserved Left Ventricular Ejection Fraction) trial of patients with HFpEF and CMR evidence of myocardial fibrosis (defined as ECV ≥ 27%), pirfenidone significantly reduced myocardial fibrosis and NT-proBNP levels compared with placebo.^148^ Although the observed magnitude of the reduction in myocardial ECV (between-group difference 2.16%) was rather small, the results indicate that reducing myocardial fibrosis via TGF-β modulation with pirfenidone may have favourable effects in HFpEF. Whether pirfenidone may improve adverse clinical outcomes and symptoms in patients with HFpEF remains to be determined by future trials.

### MicroRNA-based therapeutics

Non-coding RNAs exert a crucial role in the transcriptional and post-transcriptional regulation of fibrotic remodelling and ECM synthesis, with growing evidence suggesting specific miR as therapeutic targets to potentially revert cardiac fibrosis.^52^ MicroRNA-132 is a key modulator of signalling pathways leading to adverse cardiac remodelling and fibrosis, whereas a synthetic miR-132 inhibitor was shown to down-regulate cardiomyocyte growth and to normalize autophagy, calcium handling, and cardiac function in large animal models of LV remodelling.^151,169^ In a Phase 1b trial enrolling patients with chronic HF with LVEF between ≥30% and <50% or NT-proBNP > 125 ng, treatment with CDR132L, a specific first-in-class antisense oligonucleotide miR-132 inhibitor, resulted in a dose-dependent, sustained miR-132 reduction in plasma, while being safe and well tolerated.^150^ The currently ongoing Phase 2 HF-REVERT (Phase 2, multicentre, randomized, parallel, 3-arm, placebo-controlled Study to Assess Efficacy and Safety of CDR132L in Patients with Reduced Left Ventricular Ejection Fraction after Myocardial Infarction) trial is a proof-of-concept study to evaluate the efficacy of CDR132L in improving cardiac function and safety in patients with LVEF ≤ 45% after myocardial infarction.^149^ Another therapeutic target in cardiac fibrosis is miR-21, which is specifically up-regulated in cardiac remodelling and positively regulates cardiac fibroblast survival, aggravating cardiac fibrosis and hypertrophy as part of extracellular signal-regulated mitogen-activated protein kinase signalling.^154^ Silencing of miR-21 with specific antisense oligonucleotides has been shown to mitigate cardiac fibrosis in pressure overload-challenged mice and post-myocardial infarction HF pig models, with human *ex vivo* models of left ventricular specimens obtained from explanted hearts during transplantation surgery further confirming the involvement of miR-21 in cardiac fibrosis and antifibrotic effects of miR-21 silencing in human failing myocardium.^152–154^ Like miR-21, miR-29 is prominently expressed in cardiac myocytes, exerting an important role in cardiac remodelling by promoting pathologic hypertrophy of cardiac myocytes, whereas a global genetic deletion and a specific inhibition of miR-29 reduced cardiac hypertrophy and fibrosis and improves cardiac function in pressure overload-challenged mice.^170^ Despite the promising observed benefits on cardiac fibrosis and hypertrophy with inhibition of miR-21 and miR-29 in experimental mouse models, their efficacy in CV settings in humans remains to be evaluated.

### Experimental cell-based and epigenetic antifibrotic strategies

Besides small molecule- and miR-based therapeutics, emerging immunotherapies currently applied in the field of oncology, such as genetically modified T lymphocytes that express chimeric antigen receptors (CAR-T) technologies, may represent a promising cell-based approach to target cardiac fibroblast activation for the treatment of cardiac fibrosis.^155,171^ Indeed, initial proof-of-concept studies indicated significant reductions in cardiac fibrosis and improvements in systolic function in mouse models of CAR-T cells engineered to identify and eliminate cells expressing fibroblast activating protein (FAP) alpha.^155,156^ While CAR-T cells can be generated by various mechanisms, approaches based on reprogramming endogenous T cells to target FAP (FAPCAR) via lipid nanoparticles (LNPs) using mRNA constructs are suggested to be more safe and cost-effective compared with the adaptive transfer of exogenously derived CART-T cell therapies using retroviral vectors.^155,156^ Further research in large animal models is needed to evaluate the potential of CAR-T cell therapies for cardiac fibrosis and provide guidance for future clinical trials. Similar to CAR-T cells, regulatory T cells (Tregs) could potentially enhance cardiac remodelling by limiting inflammatory signalling and cardiac immune cell infiltration. Adoptive transfer of Tregs has been suggested to attenuate cardiac remodelling, fibrosis, and function while reducing myocardial expression of inflammatory cytokines and infiltration of inflammatory cells in mouse models of pressure overload-induced HF.^157,158^ Thus, strategies akin to CAR-T cell approaches, such as *ex vivo*-expanded Treg transfer or endogenous Treg modulation via mRNA-loaded LNPs, hold promise for future studies.^172^

Additional molecular and epigenetic antifibrotic strategies include the inhibition of cardiac fibroblast activation through monocyte-derived profibrotic inflammatory cytokines. While genetic and antibody-based ablation of resident CX3CR1^+^ tissue macrophage ablation has been shown to worsen cardiac fibrosis in murine pressure overload models, CCR2^+^ macrophages derived from circulating proinflammatory monocytes may represent a promising target to limit adverse cardiac remodelling.^159,173,174^ Evidence from small animal pressure overload models indicates that genetic or antibody-based CCR2 inhibition may reduce fibrotic cardiac remodelling.^159^ Although CCR2 antagonists are already approved for the treatment of non-cardiac conditions, their application for the management of cardiac remodelling warrants further investigation. Of note, because CCR2 is involved in acute wound healing, future studies need to identify optimal patient populations and evaluate targeted CCR2 inhibition approaches via small molecules or RNA-based techniques.^175^ Moreover, antibody-based blockade and deletion of IL-1 receptors in cardiac fibroblast were demonstrated to reduce the secretion of fibroblast-derived inflammatory cytokines and markers of cardiac fibrosis in mouse models.^160,161^ Consistently, approaches broadly inhibiting the secretome in proinflammatory macrophages have shown promising reductions in macrophage-derived IL-6 and TNFα secretion, limiting cardiac fibroblast activation, and adverse remodelling in murine pressure overload models.^162^

Beyond macrophage-based strategies, the modulation of ECM content through inhibiting fibrogenic cross-linking enzymes, such as the members of the transglutaminase family, may attenuate cardiac remodelling and hypertrophy. As such, experimental models of pressure overload indicate that pharmacological blockade of tissue transglutaminase may suppress fibrosis-associated gene expression and improve diastolic function, thereby reducing cardiomyocyte hypertrophy and fibrosis.^163^ Last, modulating stress-related epigenetic changes of cardiac fibroblast transcriptomes, such as post-translational modifications of histone tails, changes in DNA methylation, or chromatin architecture changes, may represent a promising innovative antifibrotic strategy.^166^ Potential strategies of epigenetic modulation of fibroblast activation to attenuate the development and progression of cardiac fibrosis include small molecule inhibitors of histone deacetylases, histone acetyltransferases, bromodomain extraterminal protein, and lysine demethylases.^164,165^ However, despite their therapeutic potential, genome-wide, non-specific effects of epigenetic therapies, such as side effects in non-target organs observed with pan-histone deacetylase inhibitors in cancer patients, emphasize the necessity of further identifying specific regulatory protein functions to develop targeted treatments.^176^ While experimental epigenetic antifibrotic strategies for cardiac remodelling remain the subject of preclinical development, further investigation may allow their translation into viable therapeutic options in clinical settings.

## Conclusion

Heart failure with preserved ejection fraction is characterized by phenotypical heterogeneity with a high prevalence of a broad range of cardiometabolic disorders. Comorbidities such as hypertension, obesity, or diabetes are hypothesized to contribute to the development and disease progression of HFpEF by adverse structural changes through a variety of haemodynamic and metabolic impairments. Pressure or volume overload, systemic inflammation, coronary microvascular dysfunction, capillary rarefaction, reduced nitric oxide bioavailability, and mitochondrial dysfunction may lead to adverse cardiac remodelling and myocardial fibrosis, ultimately resulting in functional impairments, such as increased diastolic stiffness or elevated filling pressures. Nearly half of all HFpEF patients exhibit LVH or concentric remodelling, which is associated with reduced exercise tolerance and a higher risk for adverse clinical events. Given the high prevalence and the important prognostic implications of LV remodelling and hypertrophy in HFpEF, specific treatment approaches directly targeting features of hypertrophy with antifibrotic agents may provide promising therapeutic benefits addressing significant unmet needs.

## Declarations

### Data Availability

No data were generated or analysed for or in support of this paper.
